# EEG microstate in obstructive sleep apnea patients

**DOI:** 10.1038/s41598-021-95749-2

**Published:** 2021-08-25

**Authors:** Xin Xiong, Yuyan Ren, Shenghan Gao, Jianhua Luo, Jiangli Liao, Chunwu Wang, Sanli Yi, Ruixiang Liu, Yan Xiang, Jianfeng He

**Affiliations:** 1 Faculty of Information Engineering and Automation, Kunming University of Science and Technology, Kunming, 650500 China; 2 College of Physics and Electronic Engineering, Hanshan Normal University, Chaozhou, 521000 China; 3 Department of Clinical Psychology, Second People’s Hospital of Yunnan, Kunming, 650021 China

**Keywords:** Neuroscience, Diseases of the nervous system, Encephalopathy, Hypoxic-ischaemic encephalopathy

## Abstract

Obstructive sleep apnea (OSA) is a common sleep respiratory disease. Previous studies have found that the wakefulness electroencephalogram (EEG) of OSA patients has changed, such as increased EEG power. However, whether the microstates reflecting the transient state of the brain is abnormal is unclear during obstructive hypopnea (OH). We investigated the microstates of sleep EEG in 100 OSA patients. Then correlation analysis was carried out between microstate parameters and EEG markers of sleep disturbance, such as power spectrum, sample entropy and detrended fluctuation analysis (DFA). OSA_OH patients showed that the microstate C increased presence and the microstate D decreased presence compared to OSA_withoutOH patients and controls. The fifth microstate E appeared during N1-OH, but the probability of other microstates transferring to microstate E was small. According to the correlation analysis, OSA_OH patients in N1-OH showed that the microstate D was positively correlated with delta power, and negatively correlated with beta and alpha power; the transition probability of the microstate B → C and E → C was positively correlated with alpha power. In other sleep stages, the microstate parameters were not correlated with power, sample entropy and FDA. We might interpret that the abnormal transition of brain active areas of OSA patients in N1-OH stage leads to abnormal microstates, which might be related to the change of alpha activity in the cortex.

## Introduction

Obstructive sleep apnea (OSA) is a common sleep disorder. With the increase of obesity rate, its prevalence is also increasing^1^. It is a chronic multisystem disease, which may lead to a variety of acute clinical problems at the same time, including hypertension, cardio cerebral stroke, mechanical infarction, etc.^2^ OSA causes repeated airflow interruption and/or reduction due to stenosis of the upper airway during sleep, resulting in fragmentation of sleep and hypoxemia^3^. These affect the neurobehavioral function of OSA patients, for example, the risk of car accident in OSA patients increases by 2–10 times^4^.

Despite the prevalence of OSA, its underlying neurophysiological process is unclear. Clinical studies have shown that the EEG of OSA patients has changed, such as changes in power spectrum and energy^5,6^. Zhou et al. found that the sample entropy of sleep apnea syndrome patients was lower than healthy controls in each sleep stage^7^. Grenèche et al. found that the power of wakefulness EEG in OSA patients after 24 h of sleep deprivation was higher than healthy controls^8^. D’Rozario et al. found that the wakefulness EEG power spectrum and detrended fluctuation analysis (DFA) of OSA patients were related to simulated driving performance, and the scale index α of DFA can be used as an indicator of simulated driving performance^9,10^. Kim et al. used DFA to analyze the sleep onset period (SOP) of narcolepsy patients, and found that the SOP of narcolepsy patients was significantly larger compared with healthy controls^11^.

These studies provide a window for us to understand OSA patients’ EEG. However, such studies are relatively few and focus on the difference of neurobehavioral ability (such as simulated driving) of OSA patients and healthy subjects^8–10^. To the best of our knowledge, there have been no reports of studying the EEG of OSA patients from the perspective of microstate. EEG microstate employs the information of the entire time and space of EEG to characterize the rapid spontaneous change of scalp potential with time^12^. Such this approach can provide a more informative framework and global interpretability without any type of a priori hypothesis^13^, in contrast with other EEG analysis techniques, which evaluate the brain’s electrical field in a specific location (for example by a priori choice of electrodes of interest) or at determinate time intervals or in specific frequency bands^14^. According to the microstate theory, EEG signals are composed of a series of topographic maps with two remarkable properties^12,15^: (1) EEG signals can be expressed with a small amount of topographic maps; (2) before the transition from one topographic map to another, a single topographic map dominates with duration of about 80–120 ms. These metastable states are microstates, which are described as the basic components or "thought atoms" of human information processing. Therefore, the microstate analysis method is more used to study human's cognition and thinking, as well as psychotic disorders^15^. However, few researchers use it to study sleep, only the healthy subjects’ and narcolepsy patients’ sleep^16,17^. Brodbeck et al. found that healthy subjects had 4 microstates during the wakefulness and NREM sleep stages^16^. Kuhn et al. found that narcoleptic patients in early NREM sleep had an additional microstate E during the N3 phase^17^. Dose the microstates of EEG in OSA patients change? If the microstates change, is there any correlation between microstate parameters and EEG markers such as power spectrum^5,6,8,9^, sample entropy^7,18^, and DFA^9–11^?

Therefore, we hypothesized that there were abnormal microstates in OSA patients during sleep obstructive apnea or obstructive hypopnea, and the microstate parameters were correlated with power spectrum, sample entropy and DFA.

## Results

### OSA of sleep stages

The stages of sleep include waking, non-rapid eye movement (N1, N2, N3), and rapid eye movement (R) ^40^. OSA may occur at any time in the sleep cycle, and the number of obstructive hypopnea (OH) occurrences in 4 sleep stages in 100 OSA patients in Subgroup_I was counted. Obstructive hypopnea occurs more frequently in N1, N2 and R stages, less in N3 stage, and least in W stages. Patients with more OH in N1 and N2 stages also have more OH in R stages, such as Sub61 and Sub80. However, patients with more OH in R stages may not have more OH in N1 and N2 stages, such as Sub45, Sub73, Sub87, Sub96, and Sub97.

### OSA microstates

**Figure 1:**
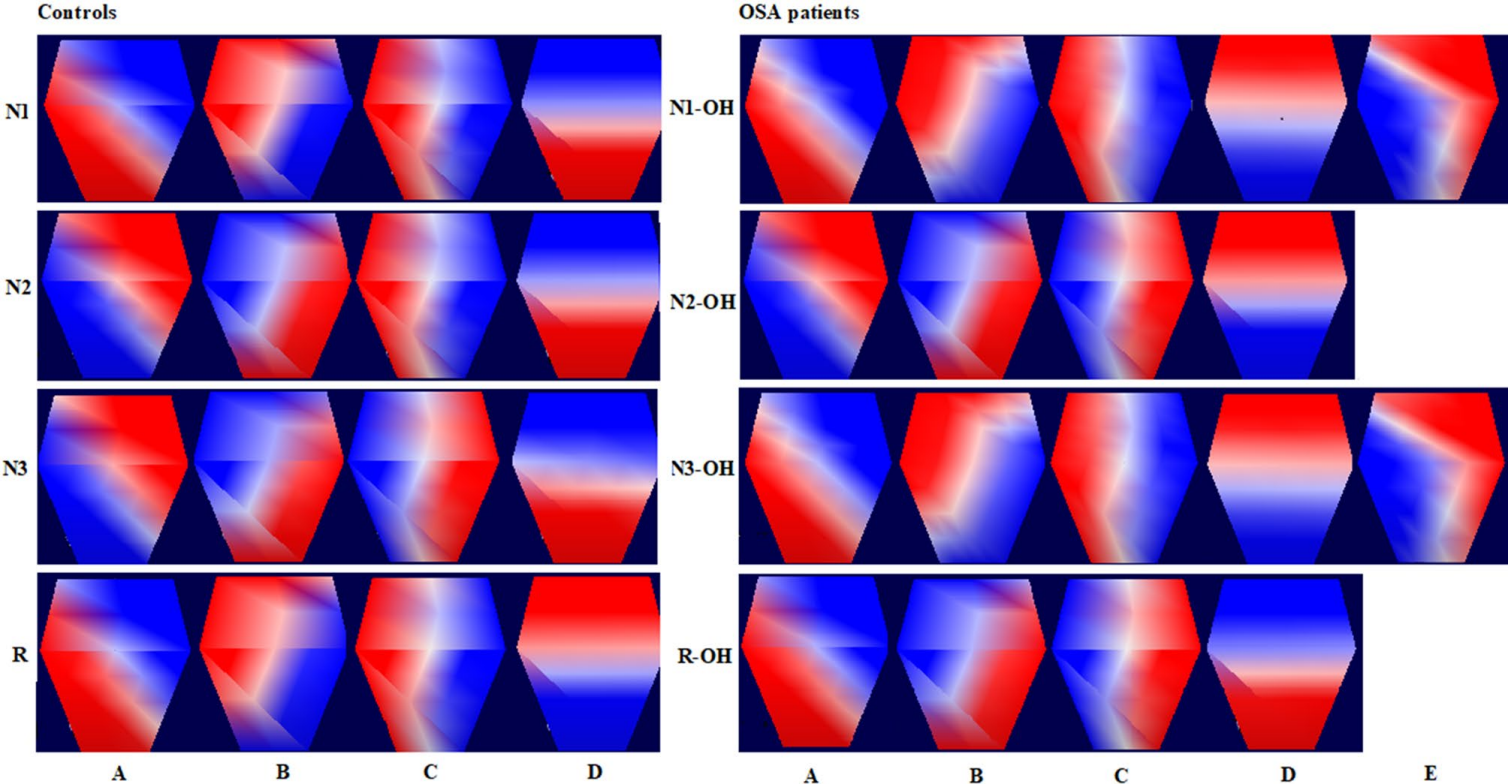
Microstates in W, N1, N2, N3 and R sleep stages in controls and OSA_OH patients.

We used CARTOOL software^19^ to estimate the microstates in OSA_OH (OSA with obstructive hypopnea) patients and controls, as shown in Fig. 1. The controls have four similar microstates A, B, C and D in N1, N2, N3 and R stages (ignoring the polarity of microstates^19,20^, which is similar to the previously reported microstate of sleep EEG^16,17^. The fifth microstate E of OSA_OH appears in N1_OH and N3_OH stages. Since there was no corresponding microstate E in controls, we made a comparison in two parts :(1) 4 typical microstates A, B, C and D were estimated respectively in OSA_OH patients and controls; (2) 5 microstates A, B, C, D and E found in N1-OH were used as topographic map templates, which were employed to fit all the sleep stages in OSA patients with and without obstructive hypopnea, that is, 5 microstates A,B,C and D and E were estimated in OSA_OH (OSA with obstructive hypopnea) and OSA_withoutOH (OSA without obstructive hypopnea) patients. Four microstates explained 71.7%, 73.4%, 76.4%, and 72.3% of the global variances in four sleep stages in OSA_OH patients, while five microstates explained 76.1%, 74.2%, 76.5%, and 72.1% of the global variances.

The calculated microstate parameters include: Global Explained Variance (Gev (%)), Mean Duration (MD (ms)), Time Coverage (TC (%)), Occurrence (Oc (/s)), and Transition Probability (TP).

The 2 × 4 × 4 (2 groups, 4 microstates, 4 microstate parameters (Gev, MD, TC, Oc)) and 2 × 5 × 4 multivariate ANOVA for each sleep stage was performed for OSA_OH vs. Control and OSA_OH vs. OSA_withoutOH, respectively, and the Bonferroni post-hoc tests were performed. The total correction models of OSA_OH vs. Control (N1: F(31,928) = 19.283, P = 0.000; N2: F(31,944) = 28.405, P = 0.000; N3: F(31,576) = 11.915, P = 0.000; R: F(31,880) = 20.430, P = 0.000) and OSA_OH vs. OSA_withoutOH (N1: F(39,2280) = 38.047, P = 0.000; N2: F(39,2296) = 28.104, P = 0.000; N3: F(39,1938) = 27.316, P = 0.000; R: F(39,2232) = 28.407, P = 0.000) showed significant differences. Then, a two-sided one-sample t test was performed on the microstate parameters within group, with significant differences indicated by stars in Tables 1, 2 and 3.

**Table 1:** Microstate parameters of 4 microstates in controls in 4 sleep stages.

	Gev (%)		MD (ms)		TC (%)		Oc/s	
	Average	SD	Average	SD	Average	SD	Average	SD
**N1**								
A	16.3	0.7	35.0	21.1	24.1	0.4	4.3*	0.3
B	16.8*	1.0	35.6	24.3	25.5	0.6	4.4	0.4
C	26.4**	0.5	35.4**	27.1	24.4**	0.3	4.3*	0.3
D	17.0	0.9	34.3	24.0	24.1**	0.3	4.3**	0.3
**N2**								
A	17.0	0.6	39.0	26.1	24.3	0.4	3.9	0.3
B	18.2	0.6	40.3	25.0	26.1**	0.5	4.1*	0.3
C	28.2**	0.7	40.6*	26.7	26.0**	0.3	4.0	0.3
D	16.1	1.0	35.9	22.8	20.9	0.5	3.7	0.3
**N3**								
A	18.5	1.2	54.5	108.0	24.7**	0.8	3.0	0.4
B	19.2	1.0	55.3	107.3	25.4	0.4	3.1	0.5
C	29.3*	0.5	55.8	98.9	25.3*	0.4	3.1	0.4
D	16.1	1.0	49.1*	98.3	20.4	0.6	2.8	0.4
**R**								
A	17.7	1.2	38.8	31.8	25.3*	0.5	4.0*	0.3
B	17.3	1.2	38.6	29.1	25.2	0.8	4.1	0.4
C	27.9*	0.4	39.7	32.0	26.2*	0.5	4.1	0.4
D	15.7	1.4	35.2	28.8	20.8*	0.5	3.7*	0.2

The stars indicate the significant difference at each sleep stage between controls and OSA-OH patients (*P < 0.05, **P < 0.01).

**Figure 2:**
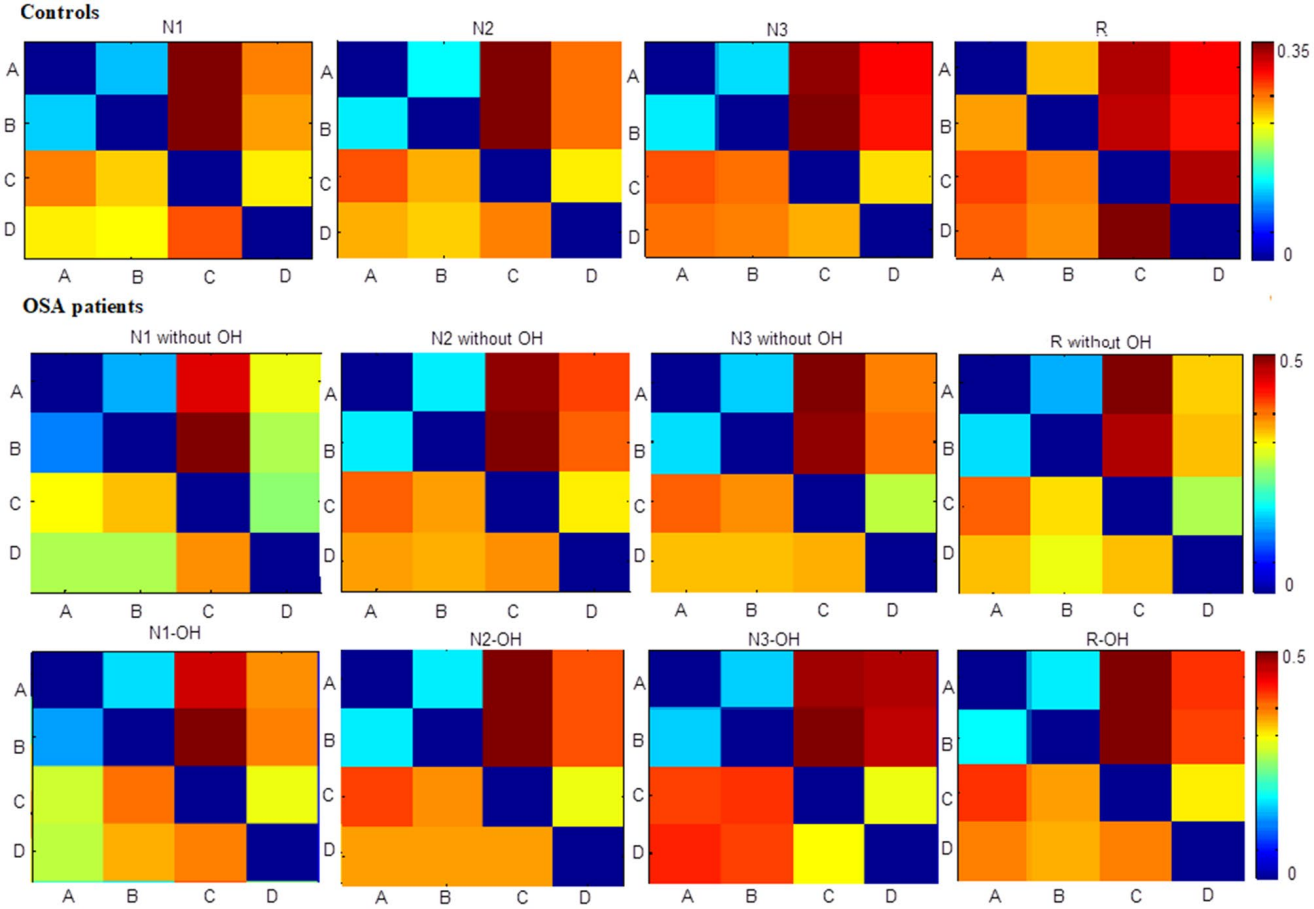
Transition probabilities (TP) of 4 microstates in controls, OSA_OH and OSA_withoutOH patients.

In Table 1, there were significant differences in parameters of microstate A, B, C and D in the four sleep stages between OSA_OH patients and controls. In Table 2, B_OC,_ C_Gev_, C_MD_, C_TC_, D_MD_, D_OC_ in N1-OH, C_Gev_, C_TC_, C_OC_, D_TC_, D_OC_ in N2-OH, A_TC_, C_TC_ in N3-OH, B_OC,_ C_Gev_, C_MD_,C_TC_, D_MD_, D_OC_ in R-OH were significantly different from those of OSA_withouOH patients. The test results of transition probability were shown in Appendix. This indicated that the microstates C and D in OSA_OH patients changed with the typical four microstate states as the fitting template, and the C_TC_ of OSA_OH was larger than that of OSA_withoueOH (N1: 0.28 ± 0.05 vs*.* 0.25 ± 0.01; N2: 0.29 ± 0.06 vs*.* 0.25 ± 0.00; N3: 0.27 ± 0.05 vs*.* 0.26 ± 0.00; R: 0.28 ± 0.05 vs*.* 0.26 ± 0.01), and the D_OC_ of OSA_OH was smaller than that of OSA_withoueOH (N1: 3.8 ± 0.01 vs*. *4.0 ± 0.00; N2: 3.5 ± 0.01 vs*.* 3.7 ± 0.00; N3: 3.5 ± 0.01 vs*.* 3.7 ± 0.00; R: 3.8 ± 0.01 vs*.* 4.0 ± 0.00). At the same time, transition probability TP_A→C_, TP_B→C_, TP_A→D_, TP_B→D_ and TP_D→C_ had significant differences in the four sleep stages. As shown in Fig. 2, the transition probability of microstates A → C, B → C, A → D, B → D, D → C in all sleep stages were relatively large.

**Table 2:** Microstate parameters of 4 microstates in OSA-OH patients in 4 sleep stages.

	Gev (%)		MD (ms)		TC (%)		Oc/s	
	Average	SD	Average	SD	Average	SD	Average	SD
**N1-OH**								
A	15.9	5.4	35.3	3.9	22.8	4.5	4.0	0.7
B	19.8	9.6	37.4	53.1	25.9	5.9	4.2*	0.5
C	20.9*	7.3	39.0*	50.0	27.7**	4.7	4.3	0.6
D	15.1	5.2	34.0*	42.0	21.1	4.0	3.8*	0.6
**N2-OH**								
A	16.6	4.1	39.0	43.3	23.5	3.4	3.8	0.5
B	17.2	4.2	40.0	47.0	24.2	3.1	3.8	0.5
C	22.7**	9.2	43.1	58.7	28.5**	5.7	4.0*	0.5
D	16.9	10.4	37.2	54.0	20.9*	4.5	3.5*	0.6
**N3-OH**								
A	16.2	6.1	50.7	98.8	20.9*	5.8	2.6	0.7
B	21.2	7.9	59.0	221.1	26.7	7.0	3.0	0.5
C	22.4	6.8	58.5	106.7	27.3*	5.1	3.0	0.5
D	16.6	7.4	50.4	131.1	21.1	5.0	2.7	0.6
**R-OH**								
A	16.3	4.8	38.4	40.5	23.8	4.2	3.8	0.6
B	19.6	10.8	32.3	45.1	24.9	5.1	3.9*	0.5
C	21.9*	10.3	41.9*	52.3	28.2*	5.8	4.1	0.5
D	14.5	3.7	35.5	34.1	20.0	2.6	3.5*	0.4

The stars indicate the significant difference at each sleep stage between OSA-OH and OSA-withoutOH patients (*P < 0.05, **P < 0.01).

In Table 3, although microstate E was only found in OSA _OH patients in N1-OH and N3- OH stages through conventional microstate calculation process, we found that there was a small amount of microstate E in each sleep stage after fitting all the sleep stages with the five microstates. Microstate E accounted for larger global variance (9%) in N1-OH. However, compared with microstates A, B, C and D during all sleep stages, microstates E occurred for shorter period of time and accounted for lower proportion of EEG signals.

**Table 3:** Microstate parameters of 5 microstates in OSA-OH patients in 4 sleep stages.

	Gev (%)		MD (ms)		TC (%)		Oc/s	
	Average	SD	Average	SD	Average	SD	Average	SD
**N1-OH**								
A	12.3	4.6	32.7	33.5	17.2	3.4	3.3*	0.6
B	20.3	10.0	37.9*	54.2	26.8	6.1	4.2	0.5
C	19.5**	7.3	37.9**	40.9	26.1**	4.6	4.2	0.5
D	15.0	5.1	33.9	38.8	21.2*	4.0	3.9*	0.6
**E**	9.0**	**2.4**	**28.4**	**35.2**	6.7**	**2.0**	1.6**	**0.2**
**N2-OH**								
A	12.5	3.7	35.4	36.1	17.5	2.7	3.2	0.5
B	17.2	4.1	40.1	42.7	24.3	3.1	3.8	0.5
C	20.7**	9.3	41.2	56.0	26.1**	5.7	3.9*	0.5
D	17.3	10.3	37.5	53.2	22.0	4.5	3.6	0.6
**E**	**6.5**	**3.7**	**30.7**	**40.2**	7.8*	**2.8**	**1.6**	**0.4**
**N3-OH**								
A	15.9	4.9	49.7	97.4	20.0	4.1	2.6	0.4
B	19.4	6.0	53.9	95.7	24.8	5.2	2.9	0.5
C	21.9*	6.3	56.6	89.0	27.0*	4.8	3.0	0.5
D	16.3	7.1	48.1	91.2	21.0	5.1	2.7	0.5
**E**	**3.3**	**1.8**	**44.5**	**117.0**	6.5**	**2.8**	1.0**	**0.4**
**R-OH**								
A	13.8*	3.7	36.4	39.4	20.1	3.4	3.4	0.5
B	19.4	10.5	39.4	45.6	24.8	5.1	3.8	0.5
C	20.8	10.6	41.4	49.5	27.2*	5.9	4.0	0.5
D	14.6*	3.7	36.0	35.4	20.7*	2.9	3.6	0.5
**E**	**3.5**	**2.0**	**33.0**	**68.0**	6.8*	**2.2**	1.3*	**0.4**

The stars indicate the significant difference at each sleep stage between OSA-OH and OSA-withoutOH patients (*P < 0.05, **P < 0.01). The bold value has significant difference, *P < 0.05, **P < 0.005.

**Figure 3:**
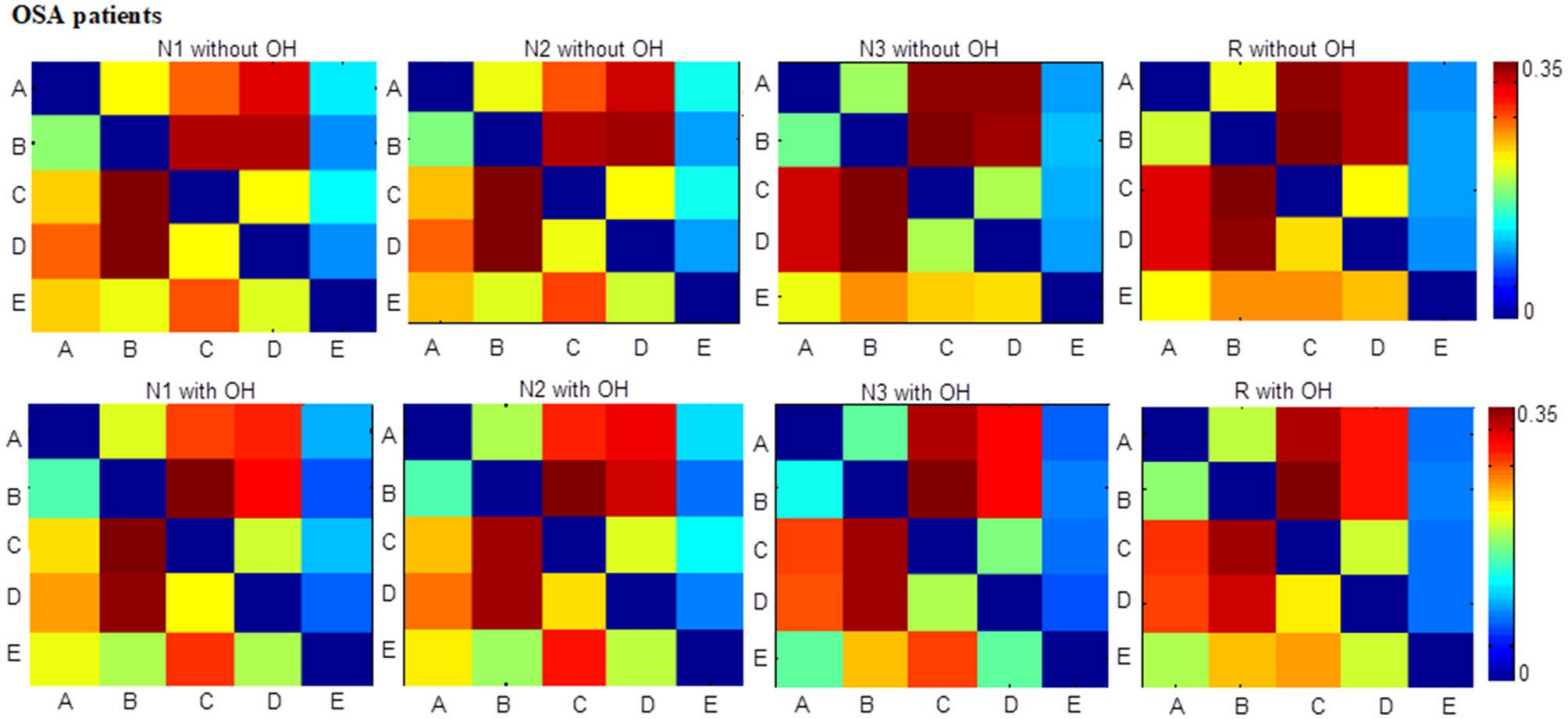
Transition probabilities (TP) of 5 microstates in OSA_OH and OSA_withoutOH patients.

In Table 3, the A_OC_, B_MD_, C_Gev_, C_MD_, C_TC_, D_TC_, D_OC_, E_Gev_, E_TC_, E_OC_ in N1-OH, C_Gev_, C_TC_, C_OC_, E_TC_ in N2-OH, C_Gev_, C_TC_, E_TC_, E_OC_ in N3-OH, A_Gev_, C_TC_, D_Gev_, D_TC_, E_OC_ in R-OH were significantly different from those of OSA_withoutOH patients. Among these parameters, it was mainly C_TC_ and E_TC_ that had changed. Compared with the results of the four microstates in Table 2, C_TC_ of the four sleep stages were all smaller, which indicated that part of the EEG segments that were originally labeled as microstate C were labeled as microstate E or other unlabeled microstates. Therefore, C_TC_ and E_TC_ had significant differences. However, the C_TC_ of OSA_OH was still larger than that of OSA_withoutOH, while the E_TC_ and E_SD_ of OSA_OH were smaller than that of OSA_withoutOH, as shown in Table 4. The transition probability from microstates A, B, D to C, E, and from microstate E to C all had significant differences, in which the transition probability from microstates A, B, D, E to C was much greater than that of E (0.31 vs. 0.10), as shown in Table 5 (only part of the transition probabilities with significant differences were given). And the transition probability from microstates A, B, D to C was greater than that of OSA_withoutOH, while the transition probability from microstates A, B, C, D to E was less than that of OSA_withoutOH, as shown in Fig. 3. This indicated that the microstates A, B, C, D and E transferred to microstates C with a high probability, and to E with a low probability after the occurrence of obstructive hypopnea.

**Table 4:** Significantly different microstate parameters C_TC_, E_TC_, Eoc of 5 microstates in OSA_OH (marked 1 in the table) and OSA_withoutOH (marked 0 in the table) patients.

Sleep stage	Groups	C_TC_	E_TC_	E_oc_
N1	1	0.26 ± 0.05	0.07 ± 0.02	0.02 ± 0.00
	0	0.24 ± 0.01	0.08 ± 0.02	0.02 ± 0.00
N2	1	0.26 ± 0.06	0.08 ± 0.03	0.02 ± 0.00
	0	0.23 ± 0.01	0.09 ± 0.01	0.02 ± 0.00
N3	1	0.27 ± 0.05	0.06 ± 0.03	0.01 ± 0.00
	0	0.24 ± 0.00	0.08 ± 0.01	0.01 ± 0.00
R	1	0.27 ± 0.06	0.07 ± 0.02	0.01 ± 0.00
	0	0.25 ± 0.01	0.07 ± 0.01	0.01 ± 0.00

**Table 5:** Significantly different transition probabilities of 5 microstates in OSA_OH and OSA_withoutOH patients (– indicates no significance in the table).

	TP_A→C_	TP_A→E_	TP_B→C_	TP_B→E_	TP_C→E_	TP_D→C_	TP_D→E_	TP_E→C_
**N1**								
1	0.31 ± 0.05	0.11 ± 0.03	0.39 ± 0.07	0.08 ± 0.03	0.12 ± 0.03	0.24 ± 0.05	0.08 ± 0.02	0.32 ± 0.06
0	0.28 ± 0.01	0.13 ± 0.02	0.34 ± 0.00	0.09 ± 0.01	0.13 ± 0.02	0.22 ± 0.02	0.10 ± 0.01	0.30 ± 0.01
**N2**								
1	0.31 ± 0.06	0.12 ± 0.04	0.37 ± 0.07	0.09 ± 0.03	0.13 ± 0.04	0.24 ± 0.06	0.09 ± 0.03	0.32 ± 0.05
0	0.28 ± 0.00	0.14 ± 0.02	0.33 ± 0.00	0.10 ± 0.00	0.14 ± 0.02	0.22 ± 0.02	0.10 ± 0.01	0.29 ± 0.01
**N3**								
1	0.38 ± 0.07	–	0.40 ± 0.09	–	0.09 ± 0.03	0.22 ± 0.05	0.08 ± 0.03	0.32 ± 0.17
0	0.35 ± 0.01	–	0.36 ± 0.01	–	0.10 ± 0.01	0.19 ± 0.02	0.10 ± 0.01	0.24 ± 0.01
**R**								
1	–	–	0.38 ± 0.06	0.09 ± 0.02	–	0.24 ± 0.04	–	–
0	–	–	0.36 ± 0.01	0.10 ± 0.01	–	0.23 ± 0.02	–	–

### Power, sample entropy and DFA of OSA patients

**Figure 4:**
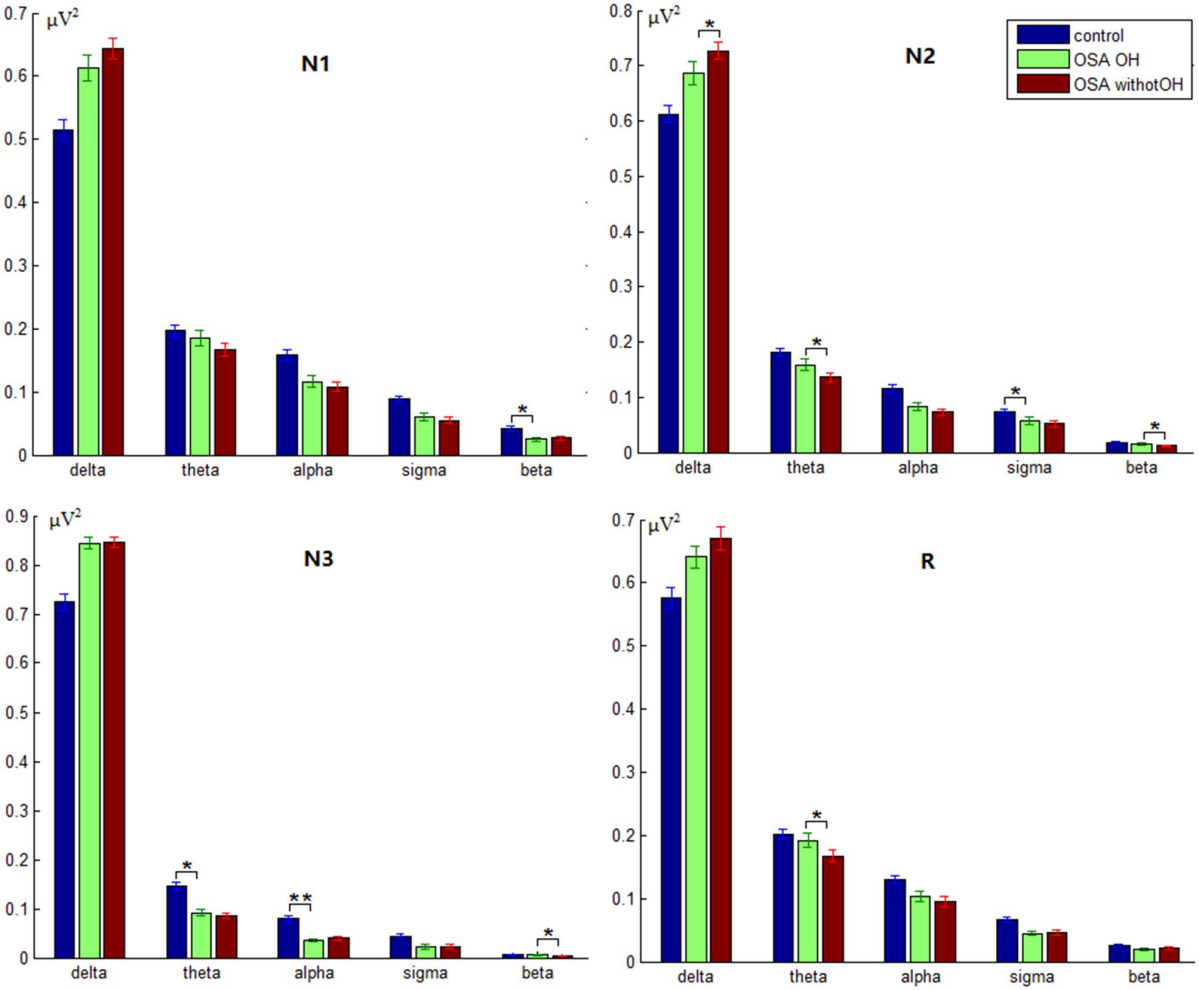
The delta, theta, alpha, sigma and beta power in four sleep stages in controls, OSA_OH and OSA_withoutOH patients ( P* < 0.05, P** < 0.05).

**Figure 5:**
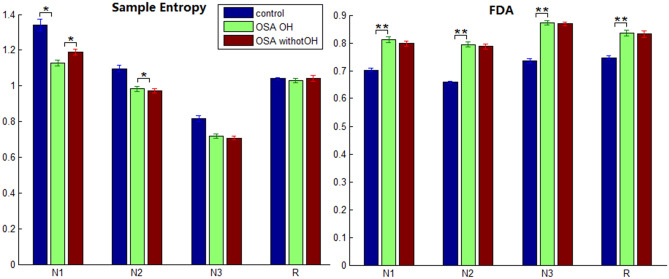
Sample entropy and FDA in four sleep stages in controls, OSA_OH and OSA_withoutOH patients (P* < 0.05, P** < 0.05).

The power, sample entropy and DFA of OSA_OH vs. Control and OSA_OH vs. OSA_withoutOH were respectively analyzed by 2 × 7 multivariate ANOVA, showing significant differences. Then, a two-sided one-sample t test was conducted within group, as shown in Figs. 4 and 5.

For OSA_OH vs. control, there was a significant difference in beta power in N1 (P = 0.026), in sigma power in N2 (P = 0.02), in delta, theta and alpha power in N3 stage (P = 0.021; P = 0.027; P = 0.007), in beta power in R (P = 0.021). For OSA_OH vs. OSA_withoutOH, there was a significant difference in beta power in N2 (P = 0.02), in beta power in N3 (P = 0.04), in theta power in R (P = 0.016).

The sample entropy of OSA_OH vs. control showed significant difference only in N1 (P = 0.015), while FDA showed significant difference in N1, N2, N3, R (P = 0.005; P = 0.001; P = 0.005; P = 0.005). The sample entropy of OSA_OH vs. OSA_withoutOH showed significant difference only in N1, N2 (P = 0.043; P = 0.042), and there was no significant difference in FDA.

### Correlation between microstate parameters and power, sample entropy and DFA

The correlation between the parameters of 4 and 5 microstates and power, sample entropy and DFA in OSA_OH and OSA_withoutOH patients was respectively compared, as shown in Tables 6 and 7.

**Table 6:** Correlation between microstate parameters of 4 microstates and power, sample entropy and DFA in OSA_OH and OSA_withoutOH patients (P < 0.05/7, i.e., 7 being the number of microstates and power, sample entropy and DFA).

OSA_OH (4 microstates)		OSA_withoutOH (4 microstates)	
	Correlation		Correlation
N1	D_MD_ − P_beta (r = − 0.362, P = 0.001)	N1	No correlation
	D_TC_ − P_beta (r = − 0.365, P = 0.007)		
	D_OC_ − P_delta (r = 0.380, P = 0.007)		
	D_OC_ − P_alpha (r = − 0.382, P = 0.006)		
	TP_B→C_ − P_alpha (r = 0.388, P = 0.005)		
N2	No correlation	N2	No correlation
N3	No correlation	N3	No correlation
R	No correlation	R	No correlation

**Table 7:** Correlation between microstate parameters of 5 microstates and power, sample entropy and DFA in OSA_OH and OSA_withoutOH patients.

OSA_OH (5 microstates)		OSA_withoutOH (5 microstates)	
	Correlation		Correlation
N1	D_OC_ − P_delta (r = 0.401, P = 0.004)	N1	TP_X→Y_ − P_delta, P_theta
	D_OC_ − P_alpha (r = − 0.376, P = 0.007)		TP_X→Y_ − SE
	TP_B→C_ − P_alpha (r = 0.486, P = 0.000)		TP_X→Y_ − FDA
	TP_E→C_ − P_alpha (r = 0.412, P = 0.003)		
N2	TP_A→B_ − FDA(r = − 0.364, P = 0.009)	N2	TP_X→Y_ − P_delta, P_theta, P_alpha
	TP_A→E_ − FDA(r = 0.396, P = 0.004)		TP_X→Y_ − FDA
N3	TP_A→E_ − FDA(r = − 0.490, P = 0.009)	N3	TP_X→Y_ − P_sigma
R	TP_C→E_ − FDA(r = 0.462, P = 0.001)	R	TP_X→Y_ − P_delta, P_theta, P_alpha,P_sigma,P_beta
			TP_X→Y_ − SE
			TP_X→Y_ − FDA

In Table 6, OSA_ withoutOH had no correlation in the four sleep stages, while OSA_OH had correlation only in N1, and only the parameters of microstate D was correlated with delta, alpha and beta power. They were positively correlated with delta power (r = 0.380, P = 0.007), and negatively correlated with alpha and beta power (r = − 0.362, P = 0.001; R = − 0.382, P = 0.006), and the TP_B→C_ was positively correlated with alpha power (r = 0.388, P = 0.005).

In Table 7, only parameters of microstate D in OSA_OH were correlated with delta and alpha power in N1, and the TP_B→C_ and TP_E→C_ was positively correlated with alpha power. The TP_A→B_ and TP_A→E_ were related to FDA in N2; TP_A→E_ were related to FDA in N3; TP_C→E_ were related to FDA in R. The five microstate parameters in OSA_withoutOH patients were all not correlated in four sleep stages, but various transition probabilities (TP_X→Y_) were correlated with delta and theta power and sample entropy (SE) and FDA in N1; TP_X→Y_ were correlated with delta, theta, alpha power and FDA in N2; TP_X→Y_ were only correlated with sigma power in N3; TP_X→Y_ were correlated with delta, theta, alpha, sigma, beta power and SE and FDA in R.

## Discussion

Microstate reflects the instantaneous state of the brain, and can identify discontinuous and nonlinear changes of global functional brain state under very high temporal resolution^12,15^. It has been found that four canonical microstates A, B, C and D are related to the activities of the posterior cingulate cortex^21^. Brodbeck et al. investigated wakefulness and NREM sleep of healthy subjects, and found that microstate C was dominant in W, N1 and N3 stages, while microstate B was dominant in N2 stage. With the increase of sleep depth, the parameter GEV of microstate D gradually decreased^16^. Kuhn et al. investigated early NREM sleep of narcoleptic patients, and found that microstate C and D were dominant in N1 stage, microstate D was still dominant in N2 and N3 stages, and an extra microstate E appeared in N3 stage^17^.

Through microstate analysis of controls, OSA_OH and OSA_withoutOH patients in 4 sleep stages, we found that the microstate C and D changed during 4 sleep periods in OSA_OH patients compared to controls and OSA_withoutOH patients. The parameter Time Coverage of microstate C increased, while Occurrence of microstate D decreased. Previous studies have shown that microstates C, D were a phenotype of schizophrenia^22^. da Cruz et al. found that patients with schizophrenia and their siblings showed increased presence of microstate class C and decreased presence of microstate class D compared to controls^23^. de Bock et al. found that microstate D was significantly decreased in psychosis in ultra-high-risk (UHR) patients with a future psychotic transition, suggesting its potential as a selective biomarker of future transition in UHR patients^24^. Kuhn et al. found that the duration of all the microstates in the N3 stage of narcoleptic patients was smaller than that of controls, and the authors believed that the microstate D of narcoleptic patients played a more important role than that of controls^17^. Microstate D was associated with attention networks according to EEG-fMRI studies^25^, so Kuhn et al. believed that the persistence of activities in the attention network of narcolepsy patients during sleep was higher^17^. Therefore, we believed that microstates C and D might also be a potential biomarker for OSA patients.

In our study, we also found that the fifth microstate E appeared in N1-OH, but the global variance of microstate E was low, only 9.0%. Although the proportion of microstate E in our study was small, it could not be considered that microstate E was caused by noise. First of all, statistical analysis showed that the parameters of microstate E (Gev (P = 0.000), TC (P = 0.000) and Oc (P = 0.000)) in OSA_OH patients were significantly different. Secondly, CARTOOL software^19^ was used to perform K-means clustering for the four sleep periods of OSA_OH and OSA_withoutoh patients. Except for the N1-OH stage, the optimal number of clustering calculated at other sleep stages was 8–15. Topographic map templates obtained from K-means clustering included four typical microstates A, B, C and D, as well as microstate E. In contrast, the optimal number of clusters in the four sleep stages in controls was always 4. When we set the number of clusters to 5–15 and checked these topographic map templates, no microstate E was found in them. Therefore, we thought that the microstate E might exist in both OSA_OH and OSA_withoutOH patients.

In the microstate calculation process, K-means clustering is a common practice, but this method has some defects in the microstate modeling^26,27^, and it may not be able to find the optimal number of clustering. In addition, there are only 6 EEG channels in ISRUC SLEEP database^28^. Although previous studies have proved that 4 typical microstate topographic maps were not limited by low spatial sampling^29^, this study only focused on 4 typical microstates, and whether the remaining microstates were affected by the number of electrodes remains to be studied. Therefore, we believed that it was necessary to expand the sample size, increase the number of electrodes, and improve the clustering method to further study the cause of the fifth microstate E in OSA patients in N1-OH.

Through correlation analysis, only parameters of microstate D in OSA_OH were correlated with delta and alpha power in N1, and the TP_B→C_ and TP_E→C_ was positively correlated with alpha power. Previous studies have shown that there was no conclusive result on the correlation between the four types of EEG microstates and specific power spectrum distribution^25,30^. However, Javed et al. believed that the uncertainty of spectral correlation of microstates involved a variety of factors, which could be eliminated by Hilbert spectral analysis^31^. The authors used Hilbert transform to transform EEG signals into delta, theta, alpha, beta, gamma bands, and then calculated the microstates in each sub-band, and the results showed that the band-wise topographies extracted using the proposed method had statistically significant similarity with full band microstates and achieved high percentage for each band in explaining EEG data variance compared to the traditional filtering method^31^. The authors also believed that an average frequency range of 10–15 Hz dominated the formation and the temporal dynamics of microstates^31^. Milz et al. investigated head-surface localization- or source-dependent power effects on the occurrence of the EEG microstate classes, and found that the EEG microstate topography was predominantly determined by intra-cortical sources in the alpha band^32^. Croce et al. investigated EEG microstates associated with intra- and inter-subject alpha variability, and observed an increase in the metrics of microstate B, with the level of intra-subject amplitude alpha oscillations, together with lower coverage of microstate D and a higher frequency of microstate C^33^. Although their study found the relationship between alpha power and microstate metrics, the authors also pointed out that there was no specificity for alpha power. The modulation effect on microstate metrics is not unique to the alpha band. It may be caused by fluctuations in other frequency bands^33^. Wegner et al. found that resting-state EEG microstates were largely determined by alpha frequencies (8–12 Hz) and microstates occur periodically with twice the alpha frequency^34^. Therefore, we believed that the intensity and spatial distribution of alpha band activity in the cortex of OSA patients changed in N1-OH, leading to changes in microstates C and D, which might also be the cause of microstates E.

Sample entropy is an improved method for measuring the complexity of time series, and it has applications in evaluating the complexity of physiological time series and diagnosing pathological state^18,35,36^. Zhou et al. found that the sample entropy of sleep apnea syndrome patients was lower than that of controls in each sleep stage^7^. We found that the sample entropy of OSA patints was significantly different from that of controls only in N1 (P = 0.015). Murphy et al. employed sample entropy to calculate the complexity of the microstate sequence over the entire template length in subjects with psychotic disorders^18^. Their results showed that there was no correlation between sequence length of microstates and entropy in psychiatric patients and controls^18^. Our study also showed that the microstate parameters were not correlated with the sample entropy.

EEG signal has a long-term correlation of dynamic oscillation characteristics^37,38^. Detrended fluctuation analysis (DFA) quantifies the time-domain fluctuation of time series by power-law method, and describes the scaling behavior or long-range correlation of time series with scale index, which is suitable for studying the correlation of long-range power-law functions of various unstable time series. Our study showed that the scale index of OSA patients and controls was 0.5 < α < 1.0, which indicated that there was a long-range power-law continuous correlation of EEG signal (with self-similarity of fractal dimension). The scale index α of OSA patients was higher than controls in four sleep stages, and the scale index α of OSA patients in N1-OH and N3-OH was higher than that of N2-OH and R-OH. D’Rozario et al. found that the DFA of the OSA patients was higher than controls with eyes opening and closing, and the DFA of the two groups with eyes opening was higher than that with eyes closing^9^. Previous studies have shown that the microstate of healthy subjects exhibit scale-free and self-similar dynamic characteristics ^39^. Murphy et al. carried out fractal analysis on the microstate of psychiatric patients, and found that the microstate sequence has a long-term time-dependent^18^. However, our study showed that the microstate parameters were not related to FDA.

However, when the five microstates were used as the fitting template, all the transition probabilities in OSA_OH patients in N1 were not correlated with sample entropy and FDA, while the transition probabilities of microstates A → B, A → E and C → E in N2, N3 and R were correlated with FDA. In OSA_withoutOH patients, there was no correlation between the five microstate parameters in the four sleep stages, but TP_X→Y_ in N1 and R stages were correlated with sample entropy, and TP_X→Y_ in N1, N2 and R stages were correlated with FDA. Our study seemed to show that there was a difference in the correlation of transition probability between OSA_OH and OSA_withoutOH patients after increasing the microstate E, but at present, there were more studies on microstate parameter (Global Explained Variance, Mean Duration, Time coverage and Occurrence^14,16–18,23,24,26,29,31–34^, but few studies on transfer probability ^17,31^.

At present, there is no simple and effective EEG biomarker that can reflect the negative impact of OSA on the brain, although D’Rozario et al. have shown that the DFA scale index has the potential as an EEG biomarker of neurobehavioral damage^9^. However, their study only compared DFA and power spectrum, and lacked comparative analysis with other EEG biomarkers (such as sample entropy, microstate, etc.). In addition, they only considered the single scene of simulated driving, and lacked the research on sleep EEG and its prognostic value. In our study, the sleep EEG of OSA patients was analyzed by the microstate method and the correlation analysis with power, sample entropy and DFA was carried out. The results showed that the microstate C increased presence and the microstate D decreased presence in OSA_OH patients. The fifth microstate E appeared during N1-OH, but the probability of other microstates transferring to microstate E was small. The microstate D in OSA_OH patients in N1-OH was correlated with delta, beta and alpha power; the transition probability of the microstate B → C and E → C was correlated with alpha power. In other sleep stages, the microstate parameters were not correlated with power, sample entropy and FDA. These showed that the microstate also had the potential as a biomarker of OSA EEG. Zappasodi el al. investigated prognostic value of EEG microstates in acute stroke, and found that a preserved microstate B in acute phase correlated with a better effective recovery^14^. Therefore, whether there is a correlation between microstate and OSA score and whether it has prognostic value for OSA patients is our next research work.

## Methods

### Data sources

The polysomnography (PSG) recordings come from an open-access sleep dataset, ISRUC-Sleep (http://sleeptight.isr.uc.pt/ISRUC_Sleep). The data were obtained from healthy subjects, subjects with sleep disorders, and subjects under the effect of sleep medication (i.e., Subgroup_I, Subgroup_II, Subgroup_III), from all-night PSG records, each with duration around 8 h, which were acquired by the Sleep Medicine Centre of the Hospital of Coimbra University (CHUC)^28^. All EEG, EOG, and EMG (chin) recordings were performed with a sampling rate of 200 Hz and stored into computer files using the standard EDF data format. The PSG recordings were composed by signals from 19 channels, of which EEG signal had 6 channels (F3, C3, O1, F4, C4 and O2). All recordings were segmented into epochs of 30 s and visually labeled by two experts according to the guidelines of AASM^40^, with the stages: awake (W), NREM (including N1, N2 and N3) and REM (abbreviated as R) sleep. Our dataset came from 30 OSA patients (excluding patients with other complications and taking medications) form Subgroup_I and 10 healthy subjects form Subgroup_III.

### EEG data pre-processing

EEG signals were pre-processed with the EEGLAB toolbox for MATLAB, which were re-referenced to the common average reference, high-pass filtered with a 0.1 Hz zero-phase FIR filter, low-pass filtered with a 45 Hz zero-phase FIR filter, and down-sampled to 100 Hz. EEG signals were inspected for artifacts with a procedure based on Independent Components (ICs) using ADJUST plug-in^41^. IC scalp maps and frequency spectra were inspected, and ICs that displayed features indicative of artifacts were removed^42^.

For OSA_OH patients, EEG epochs were extracted for each patient when labeled OH (Obstructive Hypopnea) or OA (Obstructive Apnea). For OSA_withoutOH patients, EEG epochs were extracted for each patient when labeled normal. For healthy controls, epochs were extracted from each healthy subject in each sleep stage (W, N1, N2, N3 and R). For all data, an epoch lasted for 30 s.

### Microstate analysis

Microstates reflect the instantaneous state of the brain, and can identify global functional brain states at very high temporal resolution. EEG microstates were extracted from each subject with the CARTOOL software^19^ by using a polarity-insensitive K-means algorithm in each epoch. The optimal number of microstates was determined by means of a combination of cross-validation and the Krzanovski–Lai criteria^13^. The same number of microstates was retained for each subject. The microstate maps of each subject were then submitted to a second cluster analysis in order to identify the dominant maps across the subjects^43^. Statistical smoothing was applied to remove temporally isolated topographic maps with low explanatory power. Clusters that correlated above 90% were merged, and segments shorter than 10 ms were rejected. The reference maps were selected as those that highly spatially correlated with the other maps in the same cluster. The microstate maps of each subject were matched with the reference maps showing the higher spatial correlation.

The calculated microstate parameters include: Global Explained Variance (Gev (%)), Mean Duration (MD (ms)), Time Coverage (TC (%)), Occurrence (Oc (/s)), Transition Probability (TP).

### Power spectral analysis

Previous research has shown that power spectrum analysis of wakefulness EEG is helpful to detect human's alertness^44–46^. Greneche et al. have compared the power spectrum of wakefulness EEG between OSA patients and healthy controls^8^, but we focused on the power spectrum of sleep EEG between OSA patients and healthy controls. After artefactual epochs were rejected, power spectrum was obtained using a standard fast Fourier transform (FFT) with a rectangular weighting window^47^, for each non-overlapping 5 s epoch of EEG, i.e., 500 data points ^9,10^. Absolute power spectra was calculated in the delta, theta, alpha, sigma and beta bands in each frequency ranges of 0.5–4.5, 4.5–8, 8–12, 12–15 and 15–32 Hz. Power spectrum in each sleep-staged 30 s epoch was calculated by averaging data from 6 5 s epochs. Absolute power spectrum was used to calculate to power density. For example, delta power density is equal to absolute power in the 0.5–4.5 Hz frequency range divided by the sum of absolute powers in 0.5–32 Hz frequency ranges.

### Sample entropy analysis

Sample entropy is a method to measure the complexity of time series, which has been successfully applied in the analysis of physiological signals, such as heart rate, blood pressure, EEG, etc. Its calculation results are related to the selection of parameters *m*, *r* and *n*^48^. (1) The embedding dimension *m* represents the length of the sequence. Generally, *m* is set to 1 or 2, because when m > 2, the amount of data *n* is required to be more than several thousand points^48^. (2) The physical meaning of threshold *r* is the radius of super ball with dimension *m*, which is a parameter to measure the similarity of time series, which can be set according to the needs of specific problems. Pincus believed that when *r* was set to (0.1–0.25) × SD (SD was the standard deviation), and the effective statistical characteristics could be obtained^48^. (3) The input data point *n* is set to 100–5000 in order to get effective statistical characteristics and small pseudo error for the given data. Therefore, in our study, we took *m* = 2, *r* = 0.2SD and *n* = 1000.

### Detrended fluctuation analysis

Detrended fluctuation analysis (DFA) is widely used to analyze the long-range correlation of various unsteady signals, such as ECG, EEG, DNA sequence, weather signal, turbulence velocity and temperature field. DFA is an improved root mean square analysis method, which has two advantages over the commonly used fractal analysis methods: (1) it can detect the self-similarity of time series signal that seems to be unstable but is inherently self-similarity; (2) it can avoid the obvious self-similarity trend caused by external factors^49^.

The function relationship curve of the DFA wave function *F*(*s*) and the interval length *s* is drawn in double logarithmic coordinates, then the slope of the curve by linear fitting is calculated, which is the scale index α. The scale index α provides a quantitative index for the correlation of the long-range power function. If α < 0.5, it means that the segmented time series are independent of each other; if 0.5 < α < 1.0, it means that the segmented time series have continuous correlation in the form of long-range power rate (with self-similarity of fractal dimension); if α = 1, it indicates that the segmented time series fluctuate in the form of *1*/*f* noise; if 1.0 < α < 1.5, it means that the segmented time series do not have long-range correlation; if α = 1.5, it indicates that the segmented time series are Brownian noise, that is, they are random independent. In our study, we took the data length *n* = 3000, and divided the sequence into 40 non-overlapping segments^50^.

### Statistical analysis

Multivariate ANOVA was performed for the microstate parameters, power, sample entropy and FDA of OSA_OH vs. Control and OSA_OH vs. OSA_withoutOH at each sleep stage, and Bonferroni post-hoc tests were performed. For microstate analysis, 2 × 4 × 4 and 2 × 5 × 4 multivariate ANOVA designs have been performance (2 levels: OSA_OH vs. Control or OSA_OH vs. OSA_ withoutOH), microstate (4 or 5) and Microstate parameters (Gev, MD, TC, Oc)). A two-sided one-sample t test was then performed within groups. Pearson correlation test was used to verify the correlation between microstate parameters and power, sample entropy and FDA. A threshold for significance was assessed at P < 0.00714 (i.e. 0.05/7, 7 being the number of microstates and power, sample entropy and FDA). A percentile-based bootstrap, with 1000 replicate samples, was applied to assess the 95% confidence interval of Pearson’s r values. All statistical analyses were performed using IBM SPSS statistics, version 21 (IBM Corp., Armonk, NY, USA), and P < 0.05 was considered statistically significant. All plots were performed using Matlab R2013b (MathWorks. Inc, USA) and CARTOOL software^19^.
