# Author Correction: EEG microstate in obstructive sleep apnea patients

**DOI:** 10.1038/s41598-021-00538-6

**Published:** 2021-10-21

**Authors:** Xin Xiong, Yuyan Ren, Shenghan Gao, Jianhua Luo, Jiangli Liao, Chunwu Wang, Sanli Yi, Ruixiang Liu, Yan Xiang, Jianfeng He

**Affiliations:** 1 Faculty of Information Engineering and Automation, Kunming University of Science and Technology, Kunming, 650500 China; 2 College of Physics and Electronic Engineering, Hanshan Normal University, Chaozhou, 521000 China; 3 Department of Clinical Psychology, Second People’s Hospital of Yunnan, Kunming, 650021 China

Correction to: *Scientific Reports* 10.1038/s41598-021-95749-2, published online 15 August 2021

The original version of this Article contained an error in Reference 34, which was incorrectly given as:

Fvwa, B. *et al.* EEG microstate periodicity explained by rotating phase patterns of resting-state alpha oscillations. *Neuroimage* **2**, 224 (2020).

The correct reference is listed below:

von Wegner, F. *et al*. EEG microstate periodicity explained by rotating phase patterns of resting-state alpha oscillations. *Neuroimage* **224**, 117372 (2021).

The original Article has been corrected.

